# Ultrastructure of the *Mycobacterium avium* subsp. *hominissuis* Biofilm

**DOI:** 10.1264/jsme2.ME20128

**Published:** 2021-02-17

**Authors:** Yukiko Nishiuchi

**Affiliations:** 1 Toneyama Institute for Tuberculosis Research, Osaka City University Graduate School of Medicine, 5–1–1 Toneyama, Toyonaka, Osaka 560–8552, Japan

**Keywords:** ultrastructure of biofilms, nontuberculous mycobacteria, *Mycobacterium avium* subsp. *hominissuis*, extracellular matrix, glycopeptidolipid

## Abstract

*Mycobacterium avium* subsp. *hominissuis* (MAH) is one of the most common nontuberculous mycobacterial pathogens responsible for chronic lung disease in humans. It is widely distributed in biofilms in natural and living environments. It is considered to be transmitted from the environment. Despite its importance in public health, the ultrastructure of the MAH biofilm remains largely unknown. The ultrastructure of a MAH-containing multispecies biofilm that formed naturally in a bathtub inlet was herein reported along with those of monoculture biofilms developed from microcolonies and pellicles formed in the laboratory. Scanning electron microscopy revealed an essentially multilayered bathtub biofilm that was packed with cocci and short and long rods connected by an extracellular matrix (ECM). Scattered mycobacterium-like rod-shaped cells were observed around biofilm chunks. The MAH monoculture biofilms that developed from microcolonies *in vitro* exhibited an assembly of flat layers covered with thin film-like ECM membranes. Numerous small bacterial cells (0.76±0.19‍ ‍μm in length) were observed, but not embedded in ECM. A glycopeptidolipid-deficient strain did not develop the layered ECM membrane architecture, suggesting its essential role in the development of biofilms. The pellicle biofilm also consisted of flat layered cells covered with an ECM membrane and small cells. MAH alone generated a flat layered biofilm covered with an ECM membrane. This unique structure may be suitable for resistance to water flow and disinfectants and the exclusion of fast-growing competitors, and small cells in biofilms may contribute to the formation and transmission of bioaerosols.

Nontuberculous mycobacteria (NTM) are rod-shaped, aerobic, non-motile, slow-growing, acid-fast bacteria, the cell walls of which have a high lipid content (Barksdale and Kim, 1977; Brennan and Nikaido, 1995). NTM include many species, with *Mycobacterium avium* subsp. *hominissuis* (MAH) and *Mycobacterium intracellulare*, also called the *Mycobacterium avium* complex (MAC), being the most common causes of human disease. Pulmonary MAC infections appear to be increasing in developed countries, including Japan (Field *et al.*, 2004; Namkoong *et al.*, 2016; Prevots *et al.*, 2017).

MAH and other NTM species are widely distributed; these bacteria have been recovered from water and biofilm specimens in natural and human living environments, including soil, river and pond water, drinking water distribution systems, pools, showerheads, and bathtub inlets (Vaerewijck *et al.*, 2005; Nishiuchi *et al.*, 2007; Feazel *et al.*, 2009; Nishiuchi *et al.*, 2009; Nishiuchi *et al.*, 2017). These colonizing spots in the living environment are now recognized as sources of infection. The long-term colonization of showerheads, tap water, and bathtub inlets by NTM indicates that MAH and other NTM species attach to surfaces, withstand water flow, and grow inside of plumbing pipes and fixtures as well as in bathtub inlets (Hilborn *et al.*, 2006; Nishiuchi *et al.*, 2009). These findings have led to the common understanding that NTM form biofilms.

Bacterial biofilms are generally formed through a series of steps, involving attachment to the substratum, colonization, proliferation, and maturation with an extracellular matrix (ECM). Some of the biofilms established may be dispersed to a new location (Flemming and Wingender, 2010; Chakraborty and Kumar, 2019). The bacterial ECM is composed of exopolysaccharides (EPS), proteins, lipids, and nucleic acids (Flemming and Wingender, 2010). In contrast, although mycobacteria do not produce the usual EPS components of ECM, they attach to various surfaces (Zamora *et al.*, 2007; Nishiuchi *et al.*, 2017). Mature biofilms of mycobacteria include several recognized components. Glycopeptidolipids (GPLs) are a class of amphiphilic molecules that localize to the outermost layer of the cell envelope and play a critical role in biofilm formation in *Mycolicibacterium smegmatis* and MAH (Recht and Kolter, 2001; Totani *et al.*, 2017). Extracellular DNA (eDNA) has been detected in the ECM of MAH, *M. intracellulare*, and other NTM (Rose *et al.*, 2015; Rose and Bermudez, 2016) as well as other microbes, such as *Staphylococcus* spp., *Enterococcus* spp., and *Pseudomonas aeruginosa* (Allesen-Holm *et al.*, 2006; Rice *et al.*, 2007; Thomas *et al.*, 2008). Free mycolic acids play an important role in the maturation of the pellicle biofilms of *M. tuberculosis* and *M. smegmatis* (Ojha *et al.*, 2008). Furthermore, a transposon mutant screening survey revealed that 6-oxodehydrogenase (*sucA*, a TCA cycle enzyme), protein synthetase (*pstB*, a GPL synthesis enzyme), and Rv1565c (a possible membrane protein) all play an important role in biofilm formation by *M. avium* (Freeman *et al.*, 2006; Yamazaki *et al.*, 2006).

NTM do not possess appendages, such as flagella and pili. These appendages play an important role in the first step of biofilm formation, including chemotaxis and attachment to surfaces (Hall-Stoodley *et al.*, 2004). This raises questions regarding the mechanisms by which slow-growing mycobacteria attach to surfaces and form biofilms by overcoming competition with other fast-growing microbes as well as whether mycobacteria form multispecies biofilms that contain fast-growing microbes. If this is case, the mechanisms by which mycobacteria grow inside a multispecies biofilm with other fast-growing microbes need to be elucidated. Although previous studies investigated mycobacterial biofilms (Recht and Kolter, 2001; Freeman *et al.*, 2006; Yamazaki *et al.*, 2006; Ojha *et al.*, 2008; Rose *et al.*, 2015; Rose and Bermudez, 2016; Totani *et al.*, 2017), these issues remain unclear. Furthermore, MAC may be transmitted via the ingestion of contaminated water or inhalation of aerosols directly from the environment; however, person-to-person transmission has not yet been described (Griffith *et al.*, 2007). Bathing and showering are generally presumed to be the most likely source of microbial aerosol formation (Zhou *et al.*, 2007). The mechanisms by which bioaerosols are generated at the source of infection at which mycobacterial biofilms form have not yet been elucidated. Furthermore, the structure of mycobacterial biofilms in pipes, showerheads, and bathtub inlets remains unclear. The elucidation of this structure is key to addressing the issues discussed above.

In the present study, the ultrastructure of a MAH biofilm that formed naturally on a bathtub inlet and the structure of experimentally formed monoculture biofilms were elucidated using scanning electron microscopy (SEM).

## Materials and Methods

### Environmental biofilm sampling

MAC colonies were isolated in a previous study (Nishiuchi *et al.*, 2009) from the residential bathtub inlets and showerheads of patients with pulmonary MAH infection. The biofilm specimens used in the present study were directly wiped off using a polycarbonate filter and cotton swab from a bathtub inlet from which MAH had been recovered in the previous study. The filter was subsequently processed for SEM as described below. The cotton swab was used for a culture to confirm that the biofilm specimen contained MAH as previously described (Nishiuchi *et al.*, 2009). In addition, some of the filter was observed by microscopy after Ziehl–Neelsen staining.

### MAH strains

MAH OCU806 and MAH OCU746 were isolated from the residential bathrooms of patients with pulmonary MAH infections in a previous study (Nishiuchi *et al.*, 2009). MAH OCU817 is a naturally occurring rough mutant of MAH OCU806 and GPL-deficient (Totani *et al.*, 2017).

### *In vitro* biofilm development from microcolonies

MAH strains were precultured in Middlebrook 7H9 broth (Difco) containing 0.2% (v/v) glycerol and 10% (v/v) albumin–dextrose–catalase enrichment medium (ADC; Becton, Dickinson and Co.) for 7 days. Cells were subsequently harvested and washed twice with distilled water. Cells were then re-suspended in distilled water and turbidity was adjusted to an optical density of 0.1 using the spectrophotometer mini photo 518R (TAITEC) at 660 nm, followed by a 200-fold dilution to achieve approximately 5×10^5^ colony-forming units mL^–1^ (MAH suspension). Regarding microcolony formation, 200‍ ‍μL of the MAH suspension was inoculated onto a Middlebrook 7H11-OADC agar plate (Difco) containing 0.5% (v/v) glycerol and 10% (v/v) oleic acid-ADC enrichment medium (Becton, Dickinson and Co.) and incubated at 37°C for 2 days. The microcolonies that formed were covered with coverslips (Thermanox Plastic coverslips; Thermo Fisher Scientific) for transfer and then incubated for 1 day. Coverslips with attached microcolonies were floated and cultured at 37°C for 2–3‍ ‍weeks in Middlebrook 7H9 medium without glycerol and ADC enrichment and then prepared for SEM observations.

### *In vitro* pellicle biofilm formation

Biofilms were prepared as previously reported (Totani *et al.*, 2017). Briefly, a precultured bacterial suspension was adjusted to an optical density of 0.1 and diluted 30-fold. The diluted MAH suspension was inoculated into glass tubes capped with a culture tube closure containing 2‍ ‍mL of Middlebrook 7H9 broth supplemented with 0.2% (v/v) glycerol and 10% (v/v) ADC enrichment medium and incubated at 37°C under hypoxic (5% O_2_) conditions (APM-30D; ASTEC). After 2‍ ‍weeks, the walls of the glass tubes in which pellicle biofilms had formed were prepared for SEM observations.

### SEM

Biofilms on coverslips and filters were prefixed in a solution of 2.5% glutaraldehyde in 0.1 M phosphate buffer (PB; pH 7.4) for 10‍ ‍min and rinsed three times with PB. Samples were then fixed with 2.5% glutaraldehyde for 1 h and rinsed three times with PB. Another fixation reagent, 1% (w/v) osmium tetroxide in PB, was added to the samples, followed by an incubation for 1 h. Samples were then rinsed three times with PB and dehydrated with increasing concentrations of ethanol (30, 50, 70, 90, 99, and 100%). Dehydrated samples were soaked in isoamyl acetate, critical-point-dried successively with HCP-2 (Hitachi), and coated with an 8:2 platinum–palladium alloy using an E-1030 ion sputter (Hitachi). The resultant coating had a thickness of 12 nm. Samples were observed using a S4700 scanning electron microscope (Hitachi).

### Measurement of cell sizes and statistical analysis

Bacterial rod lengths and diameters were estimated by measuring the lengths of the major and short axes of more than 50 cells. Data were compared using Wilcoxon’s rank-sum test in R ver. 3.5.2 (R core team, 2020). *P*<0.05 was considered to indicate significance.

## Results

### Biofilm structure of MAH-containing specimens

**Fig. 1.**
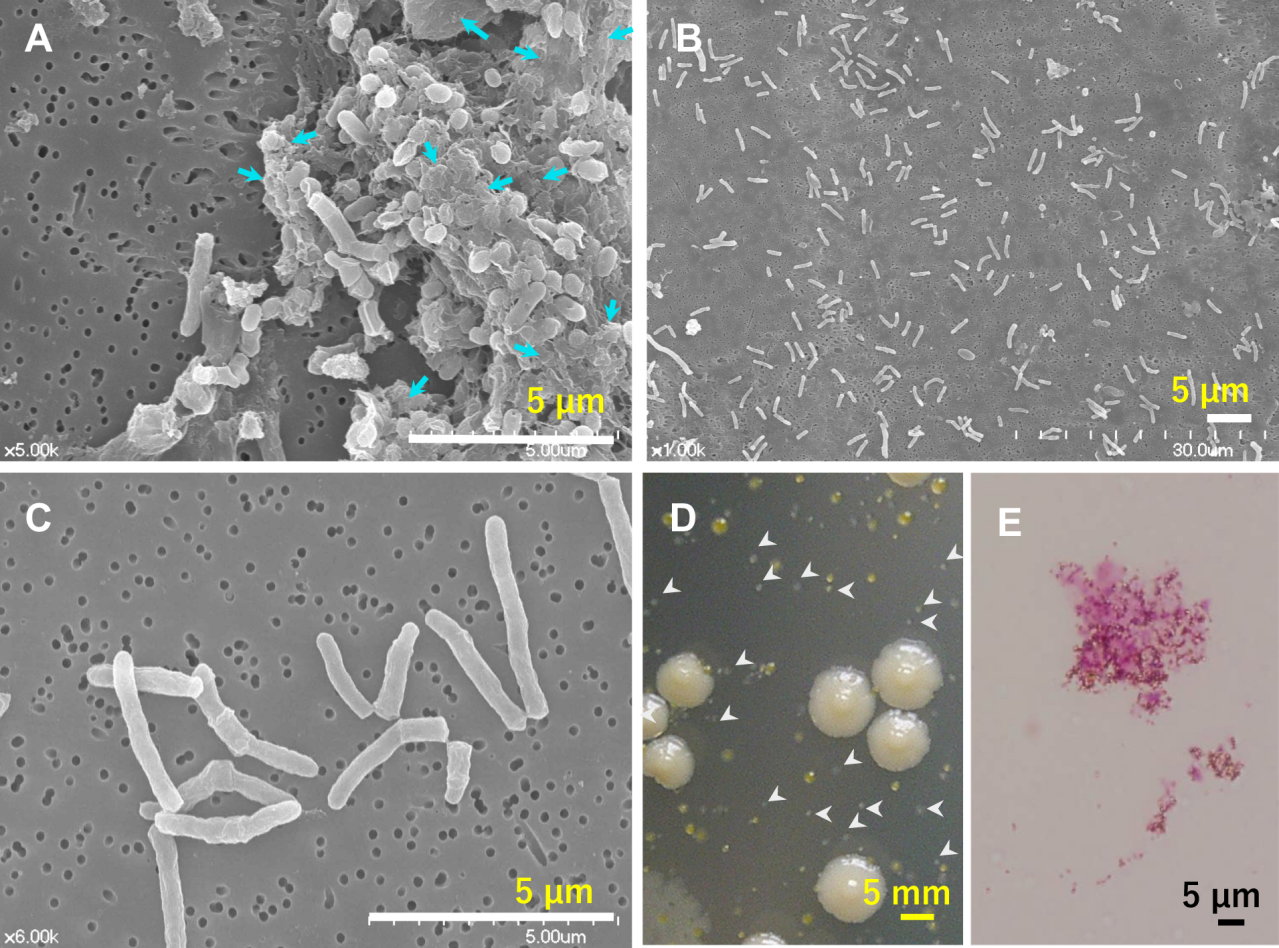
Environmental biofilms formed naturally by MAH-containing specimens on the bathtub inlet of a residential bathroom. A multilayered biofilm contains rods and cocci embedded within the ECM (blue arrows) (A). Scattered cells are rod-shaped, typical of mycobacteria-like cells (B, C). Recovered microbes isolated from MAH-containing specimens. Small and white/transparent colonies (arrowheads) were identified as MAH (D). Microscopic image of a MAH-containing specimen with Ziehl–Neelsen staining (E). Scale bars indicate 5‍ ‍μm.

The colonization of residential showerheads and bathtub inlets by MAH was previously reported (Nishiuchi *et al.*, 2007; 2009). The same genotype was observed over 3 months of colonization, suggesting that MAH biofilms had formed (Nishiuchi *et al.*, 2009). In the present study, biofilm specimens were collected by wiping the surface of a bathtub inlet with a filter and examining the ultrastructure of biofilm specimens using SEM (Fig. 1A, B, and C). The sampling site was predominantly colonized by MAH, as confirmed by culture and Ziehl–Neelsen staining (Fig. 1D and E). This naturally formed biofilm showed a multilayered structure containing cocci and short and long rods embedded within ECM (Fig. 1A). Therefore, this biofilm was composed of multiple bacterial species. On the other hand, many long rod-shaped bacterial cells, typical of mycobacterial cells, were discretely scattered in the absence of ECM on the same filter (Fig. 1B and C); the rod length was 2.32±0.82‍ ‍μm. The majority of single colony isolates obtained by culture were identified as MAH; therefore, most of these long rod-shaped cells also appeared to be MAH. Overall, MAH was not the major component in the chunks of multispecies biofilms observed. Therefore, MAH may form other types of more fragile biofilms.

### Experimentally developed MAH biofilms from microcolonies in nutrient-poor medium

**Fig. 2.**
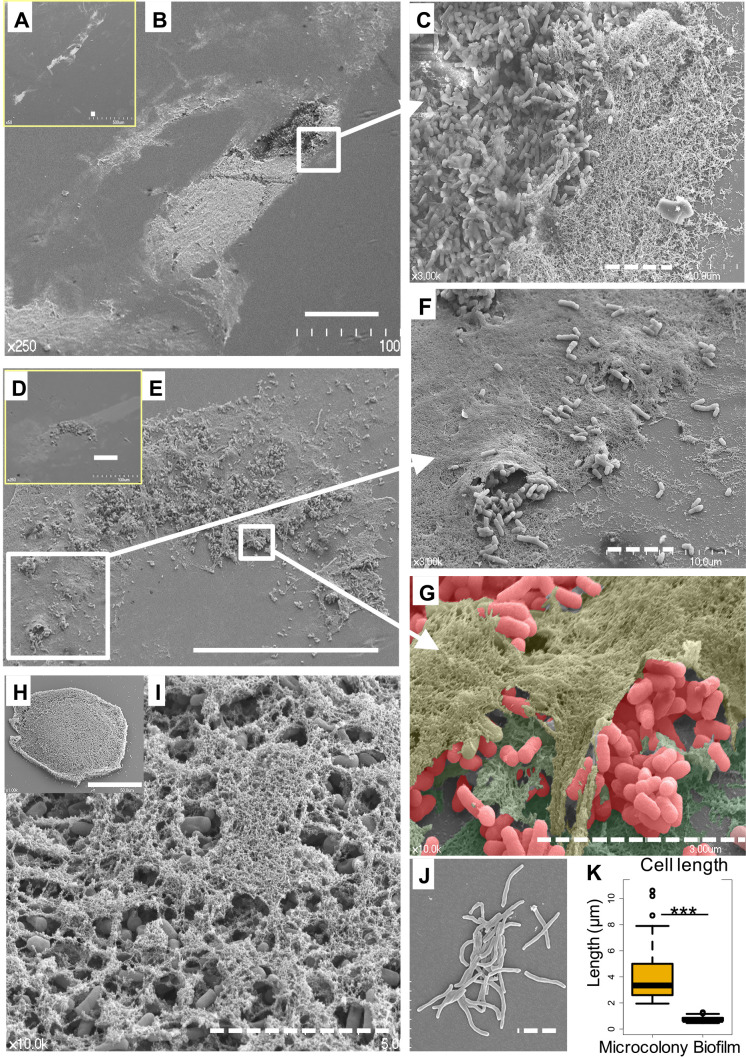
Experimentally developed MAH biofilms from microcolonies under nutrient-poor conditions. Biofilms were developed from microcolonies of MAH OCU806 for 3‍ ‍weeks (A, B, C, D, E, F, and G) and MAH OCU746 for 2‍ ‍weeks of cultivation (H, and I). Transfer of microcolonies of MAH OCU806 to a plastic coverslip before culturing (J). The solid and dashed lines of the scale bar indicate 50 and 5‍ ‍μm, respectively. The length of MAH cells in microcolonies and biofilms are represented as box plots (K). Asterisks indicate the significance of differences using the Wilcoxon rank-sum test with *** *P* < 0.0001.

To obtain insights into the structure of monoculture biofilms, MAH biofilms developed from microcolonies under nutrient-poor conditions were initially examined. The environmental strains MAH OCU806 and MAH OCU746 and the GPL-deficient strain MAH OCU817 were selected as models. Microcolonies formed on mycobacterial agar, were transferred to plastic coverslips (Fig. 2J), and cultured for 2 to 3‍ ‍weeks to develop biofilms in nutrient-poor medium. This medium was used to develop biofilms because MAH and other NTM form biofilms under nutrient-poor conditions in the environment, such as drinking water distribution systems, showerheads, and bathtub inlets (Vaerewijck *et al.*, 2005; Nishiuchi *et al.*, 2007; 2009). The OCU806 biofilm showed a flat and extended architecture (Fig. 2A, B, C, D, E, F, and G) that was approximately 100–200‍ ‍μm in length and 30–50‍ ‍μm in width. A round-shaped flat architecture, approximately 100‍ ‍μm in diameter, was observed in the SEM specimen of OCU746 (Fig. 2H). These structures of the MAH biofilm differed from the typical mushroom-shaped structure of biofilms formed by *P. aeruginosa* (Klausen *et al.*, 2003). Moreover, magnified images revealed that MAH cells were covered by a thin film-like ECM membrane (hereafter, the ECM membrane) (Fig. 2C, F, G, and I). A few bacterial cells were observed on the surface of the ECM membrane. Furthermore, biofilms were comprised of an assemblage of different ECM membranes and cells; a large number of cells was observed between, but were not embedded within ECM membranes (Fig. 2C, F, G, and I). Based on these images (Fig. 2C, F, G, and I), it was presumed that numerous MAH cells were present under ECM membranes. Cells in the biofilm were significantly shorter (0.76±0.19‍ ‍μm in length, 0.36±0.03‍ ‍μm in diameter) than those in microcolonies on coverslips on day 1 of culture (4.1±2.2‍ ‍μm in length, 0.36±0.14‍ ‍μm in diameter; *P*<0.001 in the Wilcoxon rank-sum test) (Fig. 2K). This size difference may be attributed to differences in the nutrient conditions of the culture. MAH may actively proliferate and develop biofilms even under nutrient-poor conditions. Furthermore, the GPL-deficient strain, MAH OCU817, did not develop the layered ECM membrane architecture (Fig. S1), indicating that GPL is essential for biofilm development under the culture conditions used.

### Pellicle biofilms formed by MAH

Pellicle biofilms formed at the air-liquid interface were observed using SEM (Totani *et al.*, 2017). A previous study reported that hypoxic conditions promoted the formation of a thick pellicle. However, a pellicle biofilm did not form under atmospheric or nutrient-poor conditions (Totani *et al.*, 2017). Therefore, hypoxic conditions were used to observe pellicle biofilms. We investigated the wild-type strain MAH OCU806 as a thick pellicle former and the GPL-deficient strain OCU817 as a poor pellicle former.

**Fig. 3.**
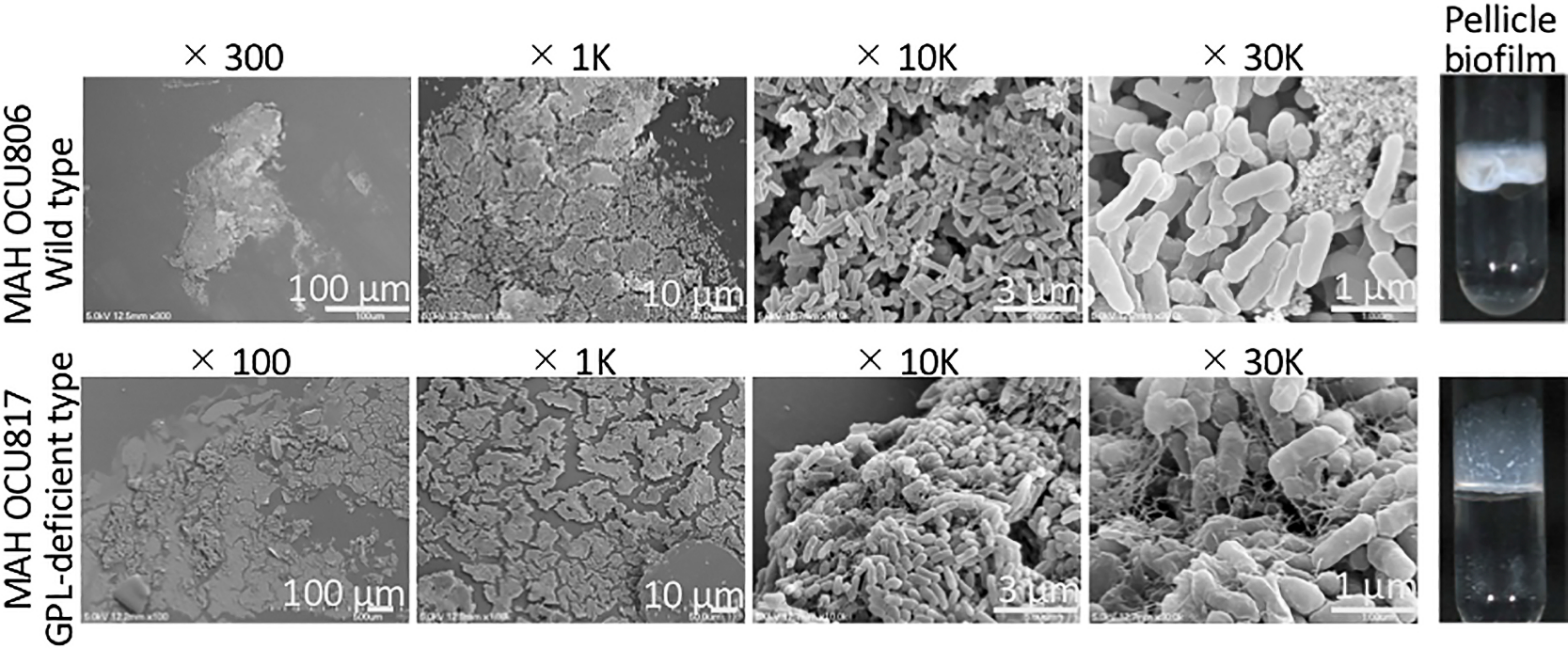
Magnification series of pellicle biofilms interacting with glass walls of test tubes. Pellicle biofilms were formed under hypoxic conditions by environmentally isolated MAH OCU806 and the naturally occurring GPL-deficient mutant strain MAH OCU817.

We focused on pellicles interacting with glass tubes (Fig. 3). Biofilms, both wild- and GPL-deficient types, on the glass wall were flat with an extended architecture possessing some ECM. The shape of ECM differed between the wild-type and GPL-deficient strains, suggesting that GPL affects the ECM ultrastructure. The former was scattered in a mat-like mass, whereas the latter was fibrous and partially covered cells in a mesh. The fibrous structure of the MAH biofilm was previously described as eDNA (Rose *et al.*, 2015; Rose and Bermudez, 2016). The fibrous material observed in the OCU817 biofilm may also be eDNA. This fibrous mesh may have fixed cells firmly to the glass wall and allowed them to grow upward. Cell sizes in both strains were smaller than 1‍ ‍μm: 0.94±0.21‍ ‍μm in length and 0.31±0.04‍ ‍μm in diameter in OCU806 and 0.92±0.15‍ ‍μm in length and 0.36±0.03‍ ‍μm in diameter in OCU817.

## Discussion

Biofilms are a multicellular form encased in a self-produced ECM (Hall-Stoodley *et al.*, 2004; Chakraborty and Kumar, 2019). The ultrastructure of a MAH-containing bathtub inlet biofilm and monoculture biofilms developed from microcolonies and pellicles were investigated to obtain a more detailed understanding of NTM biofilms. The biofilm ultrastructure of MAH (OCU806 and OCU746 in Fig. 2), *i.e.*, a flat extended cell layer covered with a thin ECM membrane, was distinct from previously reported biofilm architectures: mushroom-like (Klausen *et al.*, 2003), flat undifferentiated (Landry *et al.*, 2006), ripple-like (Stoodley *et al.*, 1999; Hall-Stoodley *et al.*, 2004), and coding morphologies (Hall-Stoodley *et al.*, 2006). In addition, an extended flat architecture was obtained in pellicle biofilms, and some parts were covered with an ECM membrane (Fig. 3). Therefore, flat layered biofilm structures are intrinsic properties of MAH. The flat and extended biofilm, as opposed to the mushroom-type biofilm of *P. aeruginosa* (Klausen *et al.*, 2003), appears to be suitable for withstanding water flow through plumbing pipes, showerheads, and bathtub inlet. The ECM membrane covers the surface of a biofilm, which also may be useful for protecting MAH cells from inhospitable environments, such as disinfectants, heat, and competition with other fast-growing microbes. NTM are tolerant of chlorine-based disinfectants (Le Dantec *et al.*, 2002), among which MAH is one of the most tolerant (Taylor *et al.*, 2000; Nishiuchi *et al.*, 2015). The formation of a MAH biofilm further increases tolerance to disinfectants (Totani *et al.*, 2017). MAH is thermoresistant (Schulze-Robbecke and Buchholtz, 1992), and the presence of MAH in hot tubs and hospital recirculating hot water systems has been associated with *M. avium* infection (von Reyn *et al.*, 1994; Kahana *et al.*, 1997). When MAH in the natural environment forms a flat layered biofilm structure similar to that observed in a monospecies culture, MAH may grow at its own pace under ECM membranes by evading survival competition with fast-growing microorganisms. This supports the ability of MAH to colonize residential bathrooms (Nishiuchi *et al.*, 2007; 2009; Arikawa *et al.*, 2019). Overall, the unique MAH biofilm structure, flat layered with an ECM membrane, may explain the behavior and survival strategies of MAH in the natural environment.

SEM images of MAH-containing bathtub biofilm specimens displayed scattered mycobacteria-like rod-shaped cells and chunks of a multilayered biofilm (Fig. 1B and C). Mycobacterial cells in biofilms may be easily dispersed. Small cell formation in experimentally formed biofilms may promote the dispersion of MAH. The small cells between ECM membranes do not adhere to each other via the ECM and may easily disperse when the ECM membrane breaks. Furthermore, this property and small-sized MAH cells may encourage the deposition of small bioaerosol particles inside lung alveoli. Shower aerosol particles were previously shown to be sufficiently small to be deposited in the deep lung (Zhou *et al.*, 2007; Brągoszewska and Biedroń, 2018).

The present study also investigated the effects of GPL on the ECM ultrastructure (Fig. 3). An essential role for GPL in the biofilm mass was reported not only for *M. avium* (Freeman *et al.*, 2006; Yamazaki *et al.*, 2006; Mukherjee and Chatterji, 2012; Totani *et al.*, 2017 MAH), but also for other GPL-producing NTM, such as *Mycobacteroides abscessus* (Howard *et al.*, 2006; Gutierrez *et al.*, 2018), *M. smegmatis* (Pang *et al.*, 2013; Pandey *et al.*, 2018), and *Mycobacterium colombiense* (Maya-Hoyos *et al.*, 2015). The ECM membrane was only observed in the wild-type strains OCU806 and OCU746 (Fig. 2). The GPL-deficient strain OCU817 did not produce an ECM (Fig. S1). The amphipathic property of GPL may help maintain the stability of the MAH biofilm. Furthermore, the eDNA mesh (Rose *et al.*, 2015) (Fig. 3) may contribute to the formation of the ECM membrane by providing a scaffold for other ECM components. In conclusion, MAH formed a flat layered biofilm structure composed of small cells covered with thin film-like ECM membranes. These results provide an insight into the behavior and survival strategies of MAH in the natural environment. Small cells in biofilms may facilitate the formation and transmission of bioaerosols.

## Citation

Nishiuchi, Y. (2021) Ultrastructure of the *Mycobacterium avium* subsp. *hominissuis* Biofilm. *Microbes Environ ***36**: ME20128.

https://doi.org/10.1264/jsme2.ME20128

## Supplementary Material

Supplementary Material
