# Supplementary material for: Ultrastructure of the Mycobacterium avium subsp. hominissuis Biofilm
Source: Microbes Environ. 2021 Feb 17;36(1):ME20128. doi: 10.1264/jsme2.ME20128 (PMC7966947; doi:10.1264/jsme2.ME20128)
Supplement: Supplementary file 1 — Supplementary Material [file 36_20128_s1.pdf]

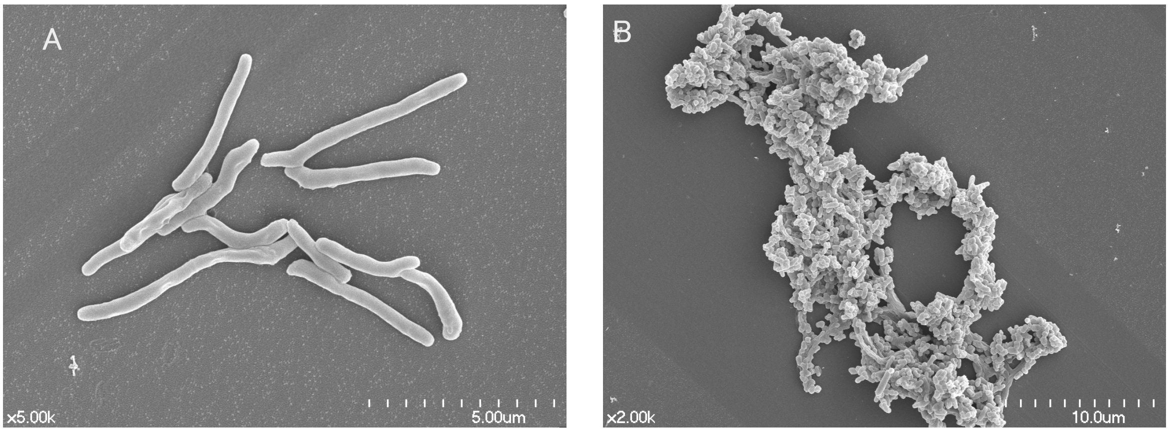

Fig. S1. Experimentally developed GPL-deficient mutant strain MAH OCU817 biofilm (B) from microcolonies (A) in nutrient-poor conditions for 2 weeks.
